# Prevalence and Prognostic Relevance of Homologous Recombination Repair Gene Mutations in Uterine Serous Carcinoma

**DOI:** 10.3390/cells11223563

**Published:** 2022-11-11

**Authors:** Lin Dong, Tingting Wang, Ning Li, Hongwen Yao, Jianming Ying, Lingying Wu, Guangwen Yuan

**Affiliations:** 1 Department of Pathology, National Cancer Center/National Clinical Research Center for Cancer/Cancer Hospital, Chinese Academy of Medical Sciences and Peking Union Medical College, Beijing 100021, China; 2 Department of Gynecology Oncology, National Cancer Center/National Clinical Research Center for Cancer/Cancer Hospital, Chinese Academy of Medical Sciences and Peking Union Medical College, Beijing 100021, China

**Keywords:** uterine serous carcinoma, homologous recombination repair, *BRCA1/2*, PARPi, prognosis

## Abstract

Uterine serous carcinoma (USC) is a rare but aggressive subtype of endometrial cancer lacking efficacious treatments. USC bears molecular and pathological resemblance to high-grade serous ovarian cancer, for which mutations in homologous recombination repair (HRR) genes have been associated with better treatment outcomes with platinum-based chemotherapy and poly-ADP ribose polymerase 1/2 inhibitors (PARPi). We aimed to investigate the prevalence of tumor HRR (tHRR) gene mutations and its potential prognostic value in USC patients. Sixty consecutive USC patients with available tumor tissue samples and complete follow-up records were included. Tumor mutations in relevant HRR genes were identified using next-generation sequencing and correlated with the progress-free survival (PFS) and disease-specific survival (DSS) of the patients. Among the 60 patients’ USC, 22 (36.7%) carried tumor HRR gene mutations (tHRRmt), with *ATM*, *BRCA1*, and *BRCA2* being the most frequently mutated genes. Survival analysis showed similar PFS (HR, 0.500; 95% CI, 0.203–1.232; *p* = 0.132), but significantly longer DSS in the tHRRmt patients than in the HRR gene wild-type (tHRRwt) patients (HR, 0.176; 95% CI, 0.050–0.626; *p* = 0.007). In FIGO stage III and IV patients, the tHRRmt group also displayed longer DSS than the tHRRwt group (*p* = 0.008). Notably, USC patients with abnormal p53 in our cohort, both PFS and DSS were significantly longer in the tHRRmt group over the tHRRwt group (*p* = 0.040 and *p* = 0.008, respectively). The HRR gene mutations are highly prevalent in USC and may be related to better clinical outcomes as a prognostic marker. Further study is needed to confirm whether tHRRmt patients may benefit from treatments targeting homologous recombination such as platinum and PARPi.

## 1. Introduction

Uterine cancer, mostly endometrial cancer (EC), is the eighth commonly diagnosed malignancy in females worldwide, with an estimated age-standardized incidence rate of 8.7 per 100,000 females in 2020 [1]. Uterine serous carcinoma (USC) is a less common but aggressive histological subtype of EC, accounting for only 5–10% of EC cases but up to 80% of EC-related deaths [2]. It is associated with poor prognosis, high recurrence rates, and low 5-year survival rates [2]. A recent study revealed a continuously increasing incidence of USC from 2001 to 2016 in contrast to that of endometroid EC, which remained stable during the same period, highlighting an urgent need for the effective treatment and management of USC [3].

Previous work by The Cancer Genome Atlas (TCGA) Research Network identified four clinically significant molecular subgroups of ECs, with USC almost exclusively falling into the copy-number high serous-like subgroup, characterized by extensive somatic copy-number alterations (SCNA) and *TP53* mutations, few *PTEN* mutations, and significantly lower progression-free survival (PFS; log-rank *p* = 0.02) compared with other EC molecular subtypes [4]. Such prognostic classification based on molecular characteristics, complemented by other clinicopathological factors, could enable a better risk assessment of EC patients [5,6]. As such, molecular analysis where *POLE*, DNA mismatch repair, and *TP53* are examined sequentially to classify ECs into the above-mentioned four subtypes is encouraged by the National Comprehensive Cancer Network (NCCN) guidelines to complement morphological assessment of the histologic tumor type [7]. Despite the integrated genomic–pathologic classification scheme, the USC subtype itself is highly heterogenic [8]. Effective management of USC patients would likely require more refined prognostic tools to potentially identify patient subpopulations that may benefit from particular treatment paradigms. 

Previous studies have revealed a molecular resemblance among USC, high-grade serous ovarian cancer (HGSOC), and basal-like breast cancer in terms of focal SCNA patterns, *TP53* mutations, low frequency of *PTEN* mutations, and pathogenic germline *BRCA1/2* mutations [4,9]. Given the many similarities, it is reasonable to hypothesize that USC may be amenable to treatment paradigms and share prognostic markers similar to those in HGSOC and breast cancer. In the latter two cancer types, homologous recombination deficiency (HRD) is associated with increased sensitivity to both the traditional, platinum-based chemotherapy and the newer therapy of poly-ADP ribose polymerase 1/2 inhibitors (PARPi) [10,11,12,13,14]. One of the contributing factors to HRD is mutations in homologous recombination repair (HRR) genes including well-known germline/somatic *BRCA1/2* mutations and mutations in other homologous recombination repair (HRR) genes such as *ATM, ATR, RAD51,* and *RAD54* [10,14,15,16]. In the case of HGSOC, large-scale cancer genome sequencing has revealed a high prevalence of HRR gene mutations [15,17], and HRR gene mutation tests and HRD assays are currently recommended to guide clinical decisions on PAPRi use in HGSOC patients [18]. As such, it is of great interest whether in USC, tHRR gene mutations can similarly serve as a prognostic indicator of patients who would benefit from treatment including chemotherapy and PARPi. Therefore, in this study, we exploited the comprehensive sequencing and clinical data of USC patients from a national cancer hospital, with the aim of systematically investigating the prevalence of tHRR gene mutations and its potential association with prognosis in USC patients.

## 2. Materials and Methods

### 2.1. Patients

Consecutive USC patients who received treatment at the Cancer Hospital of the Chinese Academy of Medical Sciences between April 2014 and June 2021 were retrospectively analyzed. For this study cohort, pathologically diagnosed USC patients with surgical tumor tissue samples and complete follow-up records available from the study center were included. Patients whose samples showed <20% tumor content in pathological examinations were excluded. Tumors were staged according to the International Federation of Gynecology and Obstetrics (FIGO) 2009 criteria. The study was approved by the ethics committee of the Cancer Hospital of the Chinese Academy of Medical Sciences.

### 2.2. Immunohistochemistry (IHC) Analysis 

One representative block of formalin-fixed, paraffin-embedded tumor tissue was selected per case. Monoclonal antibodies to p53 (clone DO-7) and mismatch repair proteins MLH1 (clone ES05), PMS2 (clone EPR3947), MSH2 (clone FE11), and MSH6 (clone EP49) (Beijing Zhongshan Golden Bridge Biotechnology, Beijing, China) were used (Supplementary Table S1). IHC staining were performed in a Ventana Benchmark IHC automated slide stainer in combination with the OptiView DAB IHC Detection Kit (Ventana Medical Systems, Arizona, United States). For MMR interpretation, the absence of nuclear staining in tumor cells, or very faint nuclear staining in focal tumor cells, was defined as a loss of protein expression (deficient mismatch repair, dMMR). p53 immunostaining was classified as normal and abnormal expression (overexpression, complete absence, and cytoplasmic staining). Overexpression is in the form of diffuse strong nuclear positivity involving at least 80% of the tumor cells. Stromal/lymphoid cells and nearby normal glandular epithelium of the bowel served as positive internal controls [19,20].

### 2.3. Genomic DNA Isolation

Formalin-fixed, paraffin-embedded tumor tissue of USC patients were collected from the pathology department biobank (Cancer Hospital of Chinese Academy of Medical Sciences, Beijing, China). Genomic DNA was extracted using a TGuide Genomic DNA One-Step Kit and a TGuide Automated Nucleic Acid Extractor (TIANGEN BIOTECH, Beijing, China) according to the manufacturer’s instructions. The quality and concentration of the DNA samples were determined with a dsDNA HS Assay Kit using a Qubit 2.0 Fluorometer (Thermo Fisher Scientific, Waltham, MA, USA). In total, 61 samples were analyzed, and one tumor sample was excluded from further analysis due to low quality. 

### 2.4. Next-Generation Sequencing Library Preparation and Sequencing

Targeted sequencing was performed using hybrid capture-based targeted next-generation sequencing. Genomic DNA was sheared using a M220 focused-ultrasonicator (Covaris LLC, Woburn, MA, USA), followed by end repairing phosphorylation and adaptor ligation (Burning Rock Biotech Ltd., Beijing, China). DNA fragments were captured using the 72-gene panel (Burning Rock Biotech Ltd., Beijing, China), which covers 370 kb of human genomic regions, and purified using the Agencourt AMPure XP Kit (Beckman Coulter, Pasadena, CA, USA). The quality and fragment size of the constructed DNA libraries were assessed by Bioanalyzer High Sensitivity DNA Analysis (Agilent Technologies Inc., Santa Clara, CA, USA). The libraries were sequenced on a Nextseq550 sequencer (Illumina Inc., San Diego, CA, USA) with pair-end reads and an average depth of at least 500-fold coverage.

### 2.5. Variant Classification and Analysis of Homologous Recombination Repair Gene Mutations

Sequencing data were aligned to the human genome (hg19) using a BWA aligner 0.7.10 [21]. Local alignment optimization and variant calling were performed using GATK 3.2 [22] and VarScan, respectively. Variants were filtered using the VarScan fpfilter pipeline. Five reads were required for each insertion–deletion mutation (INDEL), and eight reads for single-nucleotide variants (SNVs). According to the allele frequency database (ExAC, 1000 Genomes, ESP6500 etc.), variants with a frequency over 1% were considered as genetic polymorphisms [23]. Variants were annotated by ANNOVAR and SnpEff v3.6 [24,25]. DNA translocation analysis was performed using both Tophat2 and Factera 1.4.3 [26,27]. Among the customized panel, HRR genes included *ATM, ATR, BARD1, BRCA1, BRCA2, BRIP1, CDK12, CHEK1, CHEK2, FANCA, FANCD2, FANCI, FANCM, GEN1, MRE11, NBN, PALB2, RAD50, RAD51B, RAD51C, RAD51D,* and *RAD54L*, which covered the HRR genes reported in the PROfound Phase III trial (Supplementary Table S2) [28]. The clinical significance (benign/likely benign/variant of unknown significance/likely pathogenic/pathogenic) of each variant was annotated according to the guidelines of the American College of Medical Genetics and Genomics and the Association for Molecular Pathology [23]. In this study, pathogenic and likely pathogenic variants were analyzed together as HRR mutated (HRRmt), while benign, likely benign, and unknown variants were analyzed together as the HRR wild-type (HRRwt).

### 2.6. Statistical Analysis

Statistical analyses were performed using SPSS version 24 (SPSS Inc., Chicago, IL, USA). Progression-free survival (PFS) and disease-specific survival (DSS) were calculated by the Kaplan–Meier method and the differences were compared using the log-rank test and Cox proportional hazards model. Hazard ratios (HRs) with corresponding 95% CI *p* values were reported. A statistical significance was defined as a *p* value less than 0.05. 

## 3. Results

### 3.1. Clinicopathologic Characteristics

The baseline clinicopathologic characteristics are shown in Table 1. A total of 60 patients were included and followed up until November 2021, with 22 patients’ USC (36.7%) testing positive for tumor HRR gene mutations (HRRmt) and 38 (63.3%) tested negative (HRRwt). The mean age was 59.6 ± 0.9 years for the overall cohort and was 58 ± 1.9 years and 61 ± 1.1 years for the HRRmt and HRRwt patients, respectively. All patients presented with primary tumors, with the majority being FIGO stage III (43%, *n* = 26) or stage I (32%, *n* = 19). The distribution of FIGO stages was similar between the HRRmt and HRRwt groups. Half of the patients had lymphovascular space involvement, with the percentage in HRRmt patients (45%; 10/22) being slightly lower than that in HRRwt patients (53%; 20/38). Consistent with the previously identified molecular feature of USC, 85% of the included patients showed an abnormal p53 IHC, with no significant difference between the HRRmt and HRRwt groups (82% vs. 87%). One patient from each group had deficient mismatch repair (dMMR). One patient from each group had *POLE* mutation.

### 3.2. HRR Gene Mutation Profile in USC Patients

**Figure 1:**
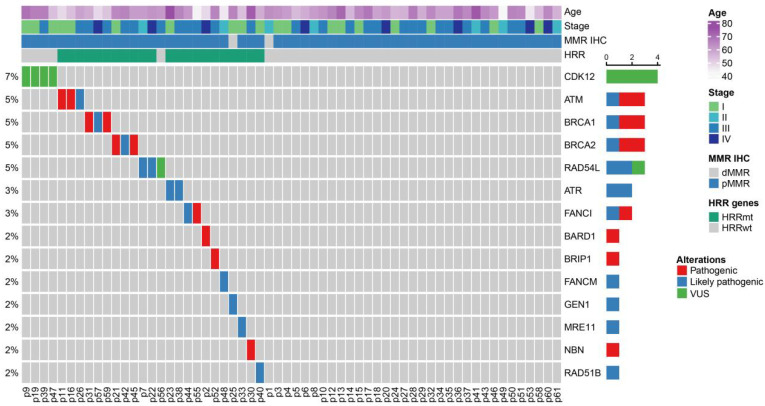
Clustered mutation profile of homologous recombination repair (HRR) genes among uterine serous carcinoma patients. All 60 patients with uterine serous carcinoma (USC) included in this study cohort were clustered according to their HRR gene mutations. The mutation profile of each patient was presented with respective baseline characteristics including age, FIGO stage, MRR IHC, and HRR gene mutation classification. The variants detected were labeled as pathogenic (red), likely pathogenic (blue), or of unknown significance (green). The prevalence of individual mutated HRR genes was calculated and presented on the left side of the figure.

The frequency of tumor HRR gene mutations was 36.7% (22/60) in the overall cohort, with 13 pathogenic or likely pathogenic HRR gene mutations identified. Aside from these, four patients in the HRRwt group were found to harbor a variant of unknown significance (VUS) in *CDK12.* The clustered HRR gene mutation profile of all 60 patients is shown in Figure 1. The most common pathogenic or likely pathogenic mutations were in *ATM*, *BRCA1*, and *BRCA2*, with mutations in each gene found in three patients (5% of the overall cohort of 60 patients), followed by *RAD54L*, *ATR*, and *FANCI*, with mutations in each gene found in two patients (3% of the overall cohort) (Figure 1; Supplementary Table S3). The other mutations detected were in the *BARD1*, *BRIP1*, *FANCM*, *GEN1*, *MRE11*, *NBN*, and *RAD51B* genes, each in a single patient. The patient with the *GEN1* mutation was the only patient with dMMR in the HRRmt group. All HRRmt patients only harbored a single gene mutation, and no co-mutation was observed. 

### 3.3. Overall Clinical Outcomes in Study Cohort

**Figure 2:**
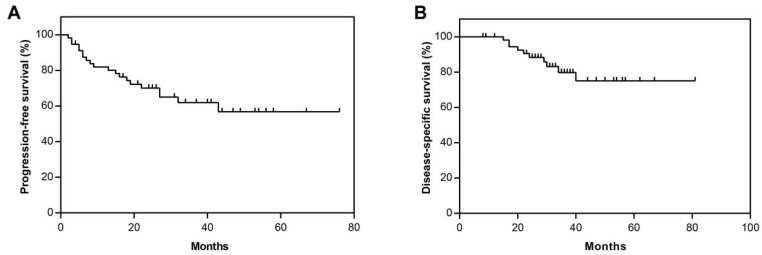
Kaplan–Meier survival analysis in the entire study cohort of uterine serous carcinomas (*n* = 60). Progression-free survival (**A**) and disease-specific survival (**B**).

For the survival analysis, the median follow-up time was 24.0 months (range: 2.0–76.0 months). One HRRmt and two HRRwt patients lacking progression and survival data were excluded from the analysis. The median PFS and DSS were not reached for the study cohort (Figure 2). Overall, there were 20 progression events and 10 death events, accounting for 35.1% and 17.5% of patients, respectively. The estimated PFS rate and DSS rate were 70.0% and 88.3% at 24 months, and 61.9% and 79.7% at 36 months, respectively.

### 3.4. Association between HRR Gene Mutations and Prognosis 

**Figure 3:**
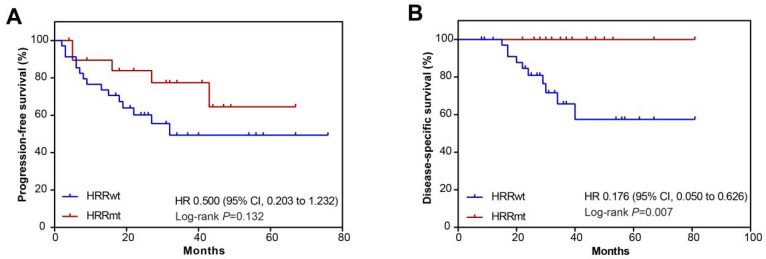
Kaplan–Meier survival analysis of molecular subgroups classified by homologous recombination repair (HRR) gene mutation status in uterine serous carcinomas from the study cohort. Progression-free survival (PFS) and disease-specific survival (DSS) are shown for each subgroup among study cohorts (**A**,**B**). HRRwt, HRR gene wild-type; HRRmt, HRR gene mutations; HR, Hazard ratios; *p* values were obtained by a two-sided log-rank test.

The PFS and DSS were compared between the HRRmt and HRRwt groups to examine the potential correlation between HRR gene mutations and the patients’ prognosis. There were five PFS events in the HRRmt group and 15 PFS events in the HRRwt group. The median PFS for the HRRwt patients was 21.5 months, while that for the HRRmt patients was not reached. Compared with the HRRwt group, the HRRmt group had numerically longer PFS despite the absence of statistical significance (HR, 0.500; 95% CI, 0.203–1.232; *p* = 0.132; Figure 3A). In terms of DSS, none of the 22 HRRmt patients died during follow-up, while 10 (27.8%) deaths occurred in the HRRwt group. The HRRmt group showed significantly longer DSS than the HRRwt group (HR, 0.176; 95% CI, 0.050–0.626; *p* = 0.007; Figure 3B). Therefore, it implied that HRRmt may serve as a prognostic biomarker for better disease-specific survival.

**Figure 4:**
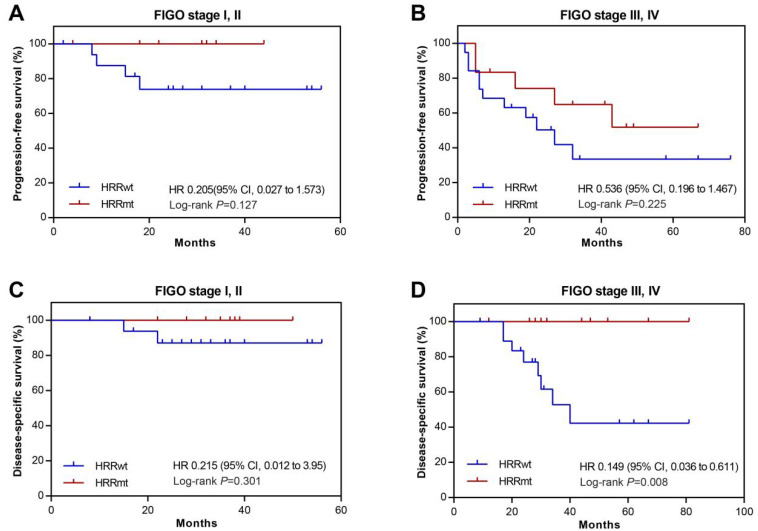
Kaplan–Meier survival analysis of subgroups classified by clinical stage among the uterine serous carcinomas from the study cohort. Progression free survival (PFS) in FIGO stages I, II (**A**) and stages III, IV (**B**); disease-specific survival (DSS) in FIGO stages I, II (**C**) and stages III, IV (**D**). HRRwt, HRR gene wild-type; HRRmt, HRR gene mutations; HR, Hazard ratios; *p* values were obtained by a two-sided log-rank test.

To obtain further insights into the association between HRR gene mutations and prognosis, we conducted subgroup analysis on patients at different FIGO stages. It was found that the frequency of HRR gene mutations was generally similar across patient subgroups stratified by tumor FIGO stage: 36.8% (7/19) for stage I, 28.6% (2/7) for stage II, 38.5% (10/26) for stage III, and 37.5% (3/8) for stage IV. Despite the numerically lower percentage of stage II patients being HRRmt, no statistically significant differences in the HRR mutation rates were found between the patient subgroups of different FIGO stages (*p* > 0.05 for all groups). The association between HRR gene mutations and prognosis was examined in early (stage I and II) and late (stage III and IV) stage patients. There was no significant difference in PFS between the HRRwt and HRRmt groups in either early- (HR, 0.205; 95% CI, 0.027–1.573; *p* = 0.127; Figure 4A) or late-stage patients (HR, 0.536; 95%, 0.196–1.467; *p* = 0.225; Figure 4B). There were two death events in early-stage patients and eight in late-stage patients, all from the HRRwt group. The difference in DSS between early-stage HRRwt and HRRmt patients did not reach statistical significance (HR, 0.215; 95% CI, 0.012–3.95; *p* = 0.301; Figure 4C). In contrast, late-stage HRRmt patients had significantly longer DSS than the HRRwt patients (HR, 0.149; 95% CI, 0.036–0.611; *p* = 0.008; Figure 4D).

### 3.5. Association between HRR Gene Mutations and Prognosis in USC Patients with Abnormal p53 Expression

**Figure 5:**
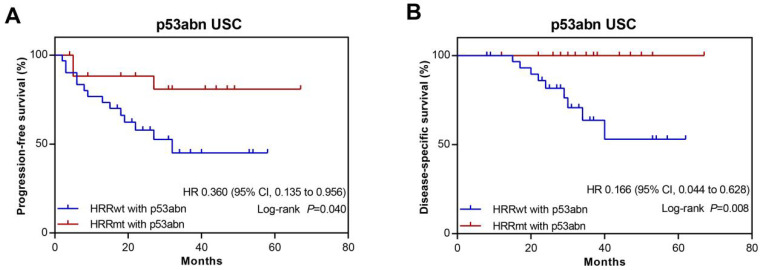
Kaplan–Meier survival analysis of molecular subgroups classified by homologous recombination repair (HRR) gene mutation status and p53abn among uterine serous carcinomas from the study cohort. Progression free survival (**A**) and disease-special survival (**B**) in patients with abnormal p53 IHC, stratified by HRR gene mutation status. p53abn, p53-abnormal; HRRwt, HRR gene wild-type; HRRmt, HRR gene mutations; HR, Hazard ratios; *p* values were obtained by a two-sided log-rank test.

According to the NCCN-recommended molecular prognostic classification scheme of ECs, USC almost exclusively falls into the *TP53*-aberrant, copy number-high subtype [7]. Given the potential link between HRR gene mutations and prognosis revealed by the analyses above, we conducted a subgroup analysis in patients with abnormal p53 IHC (p53abn) to examine whether HRR gene mutations could better stratify the prognosis of *TP53*-aberrent USC patients. There were 51 cases with p53abn among the 60 USC in the study. Twenty-three patients were diagnosed at stages I and II, and 28 were at stages III and IV. It was found that 18 patients with HRRmt among the 51 p53abn USC displayed significantly longer PFS compared with HRRwt patients with p53abn (HR, 0.360; 95% CI, 0.135–0.956; *p* = 0.040; Figure 5A and Table 1). Likewise, HRRmt patients with p53abn had significantly longer DSS compared with HRRwt patients with p53abn (HR, 0.166; 95% CI, 0.044–0.628; *p* = 0.008; Figure 5B). Taken together, tumor HRRmt is an independent biomarker with good prognosis among the USC with p53abn.

## 4. Discussion

Defects in homologous recombination, mostly caused by HRR gene mutations, could increase the risk of cancer while also sensitizing tumor cells to treatments such as platinum-based chemotherapies and PARPi [29]. As such, HRR gene mutations can serve as valuable prognostic markers to predict the response of patients to treatments targeting PARP. HRR gene mutations are prevalent among many types of cancers, especially in HGSOC [29,30]. It has been shown that in HGSOC, patients with HRR gene mutations respond better than those without to platinum-based chemotherapies and PARPi [11,12,13,14,17,31]. Previous studies have established the histological and molecular resemblance between USC and HGSOC such as poor differentiation, high frequency of copy number alterations and *TP53* mutations, and identification with BRCA1/2-associated cancer syndrome [2,4,9,17]. Therefore, whether USC also has a high prevalence of HRR gene mutations such as HGSOC and whether HRR gene mutations in USC can serve as a prognostic factor are of great interest. 

Previous studies by de Jonge et al. and Jonsson et al. reported a high prevalence of HRD phenotype and HRR gene mutations in European endometrial cancer patients but were limited by small sample sizes of USC patients among the cohorts, in which three out of four and 10 out of 19 USC were identified to be HR deficient, respectively [32,33]. In 2020, Wallbillich et al. analyzed a NGS dataset from AACR Project GENIE with a large USC cohort and presented a mutation frequency of 16.85% among the 451 USC, however, there was no clinical significance analysis associated with HRR gene mutations [34]. To obtain further insights into the prognostic implication of HRR gene mutations in USC, our study managed to assemble a relatively larger cohort of Chinese USC patients from a high-profile cancer hospital with comprehensive genomic and clinical datasets, despite the rarity of USC compared with other EC subtypes. In our cohort, 37% (22/60) of the patients carried HRR gene mutations, with *ATM*, *BRCA1*, and *BRCA2* as the most commonly mutated genes. Other mutated HRR genes included *RAD54L*, *ATR*, *FANCI*, *BARD1*, *BRIP1*, *FANCM*, *GEN1*, *MRE11*, *NBN*, and *RAD51B,* indicating a highly diverse mutation profile among the USC patients. Our study had a higher HRR gene mutation prevalence, but a similar mutation gene profile compared with the result of Wallbillich’s report [34]. The HRRwt and HRRmt groups had similar baseline characteristics such as age, FIGO stages, and lymphovascular space involvement, suggesting that the HRR mutations did not significantly affect the disease progression prior to diagnosis and treatment. Of note, HRR gene mutation presented as a promising prognostic biomarker in USC patients, showing a better survival, especially in abnormal p53 USC cases. 

Unlike endometrioid EC, USC is often associated with a worse prognosis and higher recurrence rate [2]. Differentiating patient sub-populations and tailoring anti-cancer treatments accordingly may help improve the clinical outcome of USC patients. In our study, we found that the presence of HRR mutations was associated with better DSS but not with better PFS in the whole USC cohort. Subgroup analysis further compared the PFS and DSS between the HRRwt and HRRmt groups in early- and late-stage patients and found that patients with stages III or IV USC had better DSS if carrying HRR mutations, while differences in the other comparisons did not reach statistical significance. These results likely reflect differences related to the treatment strategies before and after disease recurrence. Initially, both HRRwt and HRRmt patients with primary USC received surgery with adjuvant chemotherapy (paclitaxel combined with carboplatin) and had no obvious residual tumor. At this stage, HRR gene mutations probably did not significantly alter the risk or speed of disease progression, analogous to the above-mentioned pre-baseline situation. Consequently, the HRRwt and HRRmt patients experienced similar risks of disease recurrence with no significant difference in PFS observed, although a numerically lower recurrence rate was noted in the HRRmt group. Upon disease recurrence, most patients received chemotherapy only, and the longer DSS observed in the HRRmt group compared to the HRRwt group suggests that HRRmt patients responded to the post-recurrence chemotherapies better. This is underpinned by the magnified HRRmt-related DSS benefit observed in the late-stage patients, who likely had much poorer prognosis after chemotherapy compared with the early-stage patients. 

**Figure 6:**
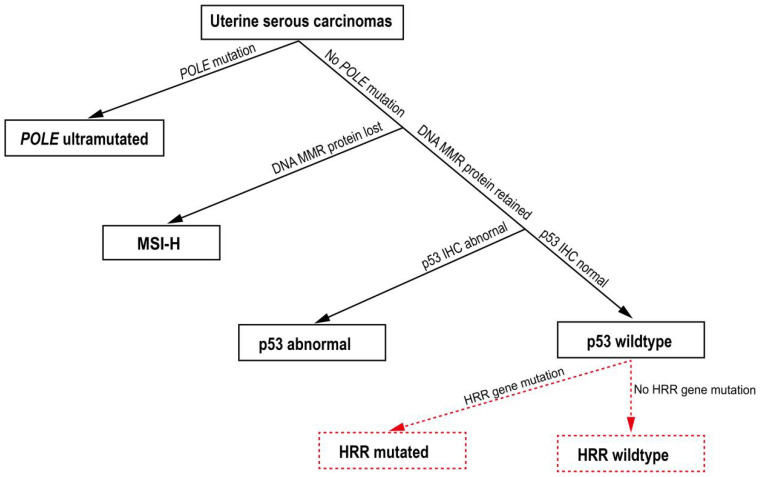
Schematic representation of the molecular classification of uterine serous carcinomas.

Importantly, our study suggested that HRR gene mutations may serve as additional prognostic markers on top of the current NCCN molecular analysis scheme of ECs. In the NCCN scheme, where *POLE*, DNA mismatch repair, and *TP53* are to be sequentially tested, USCs are mainly classified into the *TP53*-aberrant, copy number-high subtype [7]. Consistently, the majority (85%) of the patients in our cohort showed abnormal p53 IHC, and subgroup analysis on these patients revealed that both the PFS and the DSS were significantly better in the HRRmt group than in the HRRwt group. These results suggest that HRR gene mutations may provide additional, more refined prognosis within the *TP53*-aberrant, copy number-high patient population by predicting both longer overall survival and better disease control. The comprehensive molecular stratification of EC was initiated from The Cancer Genome Atlas (TCGA) project. Following this, simple and feasible classification schemes were developed using the molecular surrogates in the ProMisE and PORTEC study [35,36]. Our findings could be integrated to the current molecular classification of EC through sequentially identifying the status of *POLE*, MMR, p53, and HHR genes in USC, as illustrated in Figure 6. This was designated as the HRRmt case if being mutated in the HRR gene among the POLEwt/pMMR/p53abn USC. Taken together, our results strongly indicate that HRR gene mutations might be a valuable prognostic marker for chemotherapy in USC patients, probably in combination with other molecular markers such as *TP53*. Notably, we found no significant difference in the HRR gene mutation rate between patients at different FIGO stages, although the observed HRRmt-related survival benefit was most pronounced among the late-stage patients.

Apart from the prognostic implication in the context of chemotherapy, the sensitivity of HRR-gene-mutated HGSOC to PARPi also points to the possibility of exploring this class of anti-cancer therapies as a new treatment strategy for the hard-to-treat USC population. In fact, PARPi has shown effectiveness in not only HGSOC, but many other types of cancers carrying HRR gene mutations [10]. Aside from the most well-known *BRCA1* and *BRCA2* genes, mutations in other HRR genes have also been found to be associated with better responses to PARPi including *ATM*, *BARD1*, and *RAD51* [29]. It is thus worth exploring whether PARPi as maintenance therapy after adjuvant chemotherapy can improve the PFS and overall survival in a larger sample of USC patients.

Our study had a few limitations. First, it was a single-center study and had a relatively short follow-up period. Therefore, the impact of HRR on prognosis is not conclusive and needs further investigation. Nevertheless, the implication that HRR gene mutations can serve as a potential prognostic marker for USC patients and help identify patients that may benefit from PAPRi treatment is intriguing. Second, it should also be noted that the results reported here were limited to mutations of individual HRR genes and did not provide an integrated measure of the degree of HRD (such as an HRD score). If the correlation between HRR and USC prognosis can indeed be established in a wider USC patient population, it would then be necessary to establish an efficient and clinically relevant grading system to reliably guide the treatment of USC patients.

In conclusion, our study showed that HRR gene mutations are prevalent in Chinese USC patients. Importantly, HRRmt patients had better clinical prognosis including PFS and DSS in abnormal p53 USC. In addition, whether HRR gene mutations can serve as a prognostic marker and whether HRR mutated patients could benefit from PARPi targeted therapies still need further investigation in future study.

## Figures and Tables

**Table 1:** Clinicopathologic characteristics of uterine serous carcinoma classified by homologous recombination repair gene mutation.

	HRRmt	HRRwt	Total
Total	22 (36.7%)	38 (63.3%)	60
Age, years			
Mean ± SD	58 ± 1.9	61 ± 1.1	59.6 ± 0.9
Tumor			
Primary	22 (100%)	38 (100%)	60 (100%)
Recurrent	0 (0%)	0 (0%)	0 (0%)
FIGO stage			
I	7 (32%)	12 (32%)	19 (32%)
II	2 (9%)	5 (13%)	7 (12%)
III	10 (45%)	16 (42%)	26 (43%)
IV	3 (14%)	5 (13%)	8 (13%)
LVSI			
YES	10 (45%)	20 (53%)	30 (50%)
NO	12 (55%)	18 (47%)	30 (50%)
dMMR			
YES	1 (5%)	1 (3%)	2 (3%)
NO	21 (95%)	37 (97%)	58 (97%)
Abnormal p53 IHC			
YES	18 (82%)	33 (87%)	51 (85%)
NO	4 (18%)	5 (13%)	9 (15%)
*POLE* mutation			
YES	1 (5%)	1 (3%)	2 (3%)
NO	21 (95%)	37 (97%)	58 (97%)

Abbreviations: HRR, homologous recombination repair; mt, mutant; wt, wild-type; LVSI, lymphovascular, space involvement; dMMR, deficient mismatch repair.

## Data Availability

The data generated in this study are available within the article and its supplementary data files.

## Supplementary Materials

The following supporting information can be downloaded at: https://www.mdpi.com/article/10.3390/cells11223563/s1, Table S1: List of antibodies used for immunohistochemistry; Table S2: NGS 72-gene panel list; Table S3: HRR gene mutation profile in the study cohort.

## Novelty and Impact Statements

Our study is the first to reveal a positive association between HRR mutations and prognosis of USC patients, especially in those with abnormal p53. Our findings suggest that HRR gene mutations might enable more refined prognosis of a *TP53*-mutated USC patient subgroup in ECs when used in combination with the current TCGA molecular prognostic scheme and markers. This potential biomarker might help expand the existing molecular analysis scheme and better guide the clinical treatment decisions for USC patients. Further validation in a larger cohort would offer high accessibility to translational application in the development of clinical prognostic tools for USC.

## Abbreviations

dMMR, deficient mismatch repair; DSS, disease-specific survival; EC, endometrial cancer; HGSOC; high-grade serous ovarian cancer; HRR, homologous recombination repair; HRD, homologous recombination deficiency; HR, Hazard ratios; HRRmt, HRR gene mutations; HRRwt, HRR gene wild-type; PARPi, poly-ADP ribose polymerase 1/2 inhibitors; PFS, progress-free survival; TCGA, The Cancer Genome Atlas; USC, uterine serous carcinoma; VUS, variant of unknown significance.
