# Supplementary material for: Prevalence and Prognostic Relevance of Homologous Recombination Repair Gene Mutations in Uterine Serous Carcinoma
Source: Cells. 2022 Nov 11;11(22):3563. doi: 10.3390/cells11223563 (PMC9688566; doi:10.3390/cells11223563)
Supplement: Supplementary file 1 [file cells-11-03563-s001.zip › cells-2008746-supplementary.pdf]

Prevalence and Prognostic Relevance of Homologous Recombination Repair Gene Mutations in Uterine Serous Carcinoma

Lin Dong[1*], Tingting Wang[2*], Ning Li[2], Hongwen Yao[2], Jianming Ying[1#], Lingying Wu[2#], Guangwen Yuan[2#]

Supplementary material contents:

Table S1. List of antibodies used for immunohistochemistry

Table S2. NGS 72-gene panel list

Table S3. HRR gene mutation profile in the study cohort

Table S1. List of antibodies used for immunohistochemistry

**Primary antibody**	**Supplier**	**Type**	**Clone#**	**Cat#**	**Dilutin**
MLH1	ZSGB Bio (Beijing, China)	Monoclonal	clone ES05	ZM-0154	1:20
PMS2	ZSGB Bio	Monoclonal	clone EPR3947	ZA-0542	1:20
MSH2	ZSGB Bio	Monoclonal	clone FE11	TA347005	1:20
MSH6	ZSGB Bio	Monoclonal	clone EP49	ZA-0541	1:20
p53	ZSGB Bio	Monoclonal	clone DO-7	ZM-0408	1:20

Table S2. NGS 72-gene panel list

Gene	Gene	Gene	Gene	Gene
**ABRAXAS1** NM_139076.2	**AKT1** NM_001014432.1	**APC** NM_000038.5	**AR** NM_000044.3	**ATM** NM_000051.3
**ATR** NM_001184.3	**BAP1** NM_004656.3	**BARD1** NM_000465.3	**BRAF** NM_004333.4	**BRCA1** NM_007294.3
**BRCA2** NM_000059.3	**BRIP1** NM_032043.2	**CCND1** NM_053056.2	**CDK12** NM_016507.3	**CDKN1B** NM_004064.4
**CDKN2A** NM_000077.4	**CDKN2B** NM_004936.3	**CHD1** NM_001270.2	**CHEK1** NM_001274.5	**CHEK2** NM_007194.3
**CTNNB1** NM_001904.3	**EMSY** NM_001300942.1	**EPCAM** NM_002354.2	**ERCC2** NM_000400.3	**ERCC3** NM_000122.1
**ERCC4** NM_005236.2	**ESR1** NM_000125.3	**FANCA** NM_000135.2	**FANCD2** NM_001018115.2	**FANCI** NM_001113378.1
**FANCL** NM_018062.3	**FANCM** NM_020937.3	**FOXA1** NM_004496.3	**GEN1** NM_001130009.2	**HDAC2** NM_001527.3
**HOXB13** NM_006361.5	**MLH1** NM_000249.3	**MLH3** NM_001040108.1	**MRE11** NM_005591.3	**MSH2** NM_000251.2
**MSH6** NM_000179.2	**MUTYH** NM_001128425.1	**MYC** NM_002467.4	**NBN** NM_002485.4	**NCOR1** NM_006311.3
**NCOR2** NM_006312.5	**PALB2** NM_024675.3	**PIK3CA** NM_006218.3	**PIK3CB** NM_006219.2	**PIK3R1** NM_181523.2
**PMS2** NM_000535.6	**POLE** NM_006231.3	**PPP2R2A** NM_002717.3	**PTEN** NM_000314.6	**RAD50** NM_005732.3
**RAD51** NM_001164269.1	**RAD51B** NM_133509.3	**RAD51C** NM_058216.2	**RAD51D** NM_002878.3	**RAD52** NM_001297419.1
**RAD54L** NM_003579.3	**RAF1** NM_002880.3	**RB1** NM_000321.2	**RNF43** NM_017763.5	**RSPO2** NM_178565.4
**SPOP** NM_001007226.1	**STK11** NM_000455.4	**TMPRSS2** NM_001135099.1	**TP53** NM_000546.5	**XRCC2** NM_005431.1
**ZBTB16** NM_001018011.1	**ZNRF3** NM_001206998.1			

Table S3. HRR gene mutation profile in the study cohort

Case ID	NGS ID	Age	FIGO Stage	MMR status	Gene	Mutation_Type	Exon_R	HGVS_C	HGVS_P	AF	Degrees	HRR mutation	Progression-free survival (months)	Disease-specific survival	Status (1: progression; 0: normal)	Survival status(1:died; 0: live)
1	AQ1902425FFP	63	II	dMMR								HRRwt	17	22	0	1
2	AQ1902426FFP	48	IV	pMMR	BARD1	frameshift_variant	3	c.292_295del	p.Asn98fs	30.04%	Pathogenic	HRRmt	16	28	1	0
3	AQ1902427FFP	62	I	pMMR								HRRwt	31	36	0	0
4	AQ1902428FFP	60	I	pMMR								HRRwt	27	27	0	0
5	AQ1902429FFP	54	III	pMMR								HRRwt	21	27	0	0
6	AQ1902430FFP	57	IV	pMMR								HRRwt	6	24	1	1
7	AQ1902431FFP	60	II	pMMR	RAD54L	missense_variant	15	c.1624C>T	p.Arg542Cys	48.98%	Likely pathogenic	HRRmt	22	28	0	0
8	AQ1902432FFP	60	II	pMMR								HRRwt	31	31	0	0
9	AQ1902433FFP	69	I	pMMR	CDK12	cn_amp	NA	cn_amp	NA	26.3	VUS	HRRwt	18	33	1	0
10	AQ1902434FFP	62	III	pMMR								HRRwt	34	34	0	0
11	AQ1902435FFP	48	I	pMMR	ATM	stop_gained	13	c.1921G>T	p.Glu641*	18.07%	Pathogenic	HRRmt	31	38	0	0
12	AQ1902436FFP	65	I	pMMR								HRRwt	40	40	0	0
13	AQ1902437FFP	71	I	pMMR								HRRwt	54	54	0	0
14	AQ1902438FFP	55	III	pMMR								HRRwt	21	28	0	0
15	AQ1902439FFP	64	I	pMMR								HRRwt	15	23	1	0
16	AQ1902440FFP	53	I	pMMR	ATM	stop_gained	7	c.748C>T	p.Arg250*	16.00%	Pathogenic	HRRmt	18	22	0	0
17	AQ1902441FFP	68	III	pMMR								HRRwt	6	23	1	0
18	AQ1902442FFP	59	III	pMMR								HRRwt	3	17	1	1
19	AQ1902443FFP	66	I	pMMR	CDK12	cn_amp	NA	cn_amp	NA	26.3	VUS	HRRwt	24	25	0	0
20	AQ1902444FFP	61	IV	pMMR								HRRwt	2	20	1	1
21	AQ1902445FFP	64	I	pMMR	BRCA2	frameshift_variant	10	c.956dup	p.Asn319fs	50.21%	Pathogenic	HRRmt	32	35	0	0
22	AQ1902446FFP	62	IV	pMMR	RAD54L	missense_variant	15	c.1624C>T	p.Arg542Cys	44.60%	Likely pathogenic	HRRmt	27	44	1	0
23	AQ1902447FFP	76	I	pMMR	ATR	frameshift_variant	4	c.589_591delinsC	p.Met197fs	7.23%	Likely pathogenic	HRRmt	31	37	0	0
24	AQ1902448FFP	55	I	pMMR								HRRwt	17	23	0	0
25	AQ1902507FFP	65	I	dMMR	GEN1	frameshift_variant	2	c.130del	p.Met44fs	14.63%	Likely pathogenic	HRRmt	34	39	0	0
26	AQ2003074FFP	59	III	pMMR	ATM	frameshift_variant	40	c.5977dup	p.Ser1993fs	18.34%	Likely pathogenic	HRRmt	43	81	1	0
27	AQ2003076FFP	56	III	pMMR								HRRwt	76	81	0	0
28	AQ2003075FFP	67	III	pMMR								HRRwt	7	17	1	1
29	AQ2003077FFP	57	III	pMMR								HRRwt	67	67	0	0
30	AQ2003078FFP	73	III	pMMR	NBN	frameshift_variant	2	c.93_94del	p.Ala32fs	34.90%	Pathogenic	HRRmt	67	67	0	0
31	AQ2003079FFP	57	III	pMMR	BRCA1	stop_gained	10	c.2722G>T	p.Glu908*	56.39%	Pathogenic	HRRmt	67	67	0	0
32	AQ2003080FFP	64	I	pMMR								HRRwt	8	15	1	1
33	AQ2003081FFP	53	I	pMMR	MRE11	frameshift_variant	14	c.1545del	p.Asp516fs	52.65%	Likely pathogenic	HRRmt	4	8	0	0
34	AQ2003085FFP	59	III	pMMR								HRRwt	Unknown	Unknown	Unknown	Unknown
35	AQ2003082FFP	59	III	pMMR								HRRwt	3	9	1	0
36	AQ2003083FFP	68	IV	pMMR								HRRwt	27	34	1	1
37	AQ2003084FFP	64	III	pMMR								HRRwt	58	62	0	0
38	AQ2003086FFP	65	III	pMMR	ATR	frameshift_variant	20	c.3764del	p.Leu1255fs	37.44%	Likely pathogenic	HRRmt	Unknown	Unknown	Unknown	Unknown
39	AQ2003087FFP	66	III	pMMR	CDK12	cn_amp	NA	cn_amp	NA	7	VUS	HRRwt	19	30	1	1
40	AQ2003088FFP	57	I	pMMR	RAD51B	large_genomic_rearrange	6-11	exon6-11cn_amp	NA	4	Likely pathogenic	HRRmt	44	50	0	0
41	AQ2003089FFP	74	II	pMMR								HRRwt	56	56	0	0
42	AQ2003090FFP	65	III	pMMR	BRCA2	missense_variant	19	c.8350C>T	p.Arg2784Trp	2.16%	Likely pathogenic	HRRmt	47	53	0	0
43	AQ2003091FFP	64	III	pMMR								HRRwt	22	40	1	1
44	AQ2003092FFP	61	III	pMMR	FANCI	stop_gained	19	c.1840C>T	p.Arg614*	3.08%	Likely pathogenic	HRRmt	49	53	0	0
45	AQ2003093FFP	60	III	pMMR	BRCA2	splice_acceptor_variant	2	c.-39-1_-39del	NA	49.73%	Pathogenic	HRRmt	41	47	0	0
46	AQ2003095FFP	61	I	pMMR								HRRwt	37	37	0	0
47	AQ2003096FFP	53	I	pMMR	CDK12	cn_amp	NA	cn_amp	NA	4.4	VUS	HRRwt	53	53	0	0
48	AQ2003104FFP	45	II	pMMR	FANCM	stop_gained	1	c.267C>A	p.Cys89*	11.50%	Likely pathogenic	HRRmt	32	32	0	0
49	AQ2003109FFP	42	II	pMMR								HRRwt	25	29	0	0
50	AQ2003108FFP	65	III	pMMR								HRRwt	15	29	0	1
51	AQ2003105FFP	64	III	pMMR								HRRwt	26	31	0	0
52	AQ2003103FFP	65	III	pMMR	BRIP1	stop_gained	5	c.484C>T	p.Arg162*	84.21%	Pathogenic	HRRmt	32	32	0	0
53	AQ2003097FFP	45	IV	pMMR								HRRwt	Unknown	Unknown	Unknown	Unknown
54	Not done	43	I	pMMR								ND(QC failed	Unknown	43	0	0
55	AQ1901682FFP	43	III	pMMR	FANCI	stop_gained	24	c.2593C>T	p.Gln865*	48%	Pathogenic	HRRmt	5	30	1	0
56	AQ1904412FFP	61	III	pMMR	RAD54L	fusion	Exon19	Exon19_Intron12	RAD54L-MAST2	36%	VUS	HRRwt	13	23	1	0
57	AQ2000997FFP	48	IV	pMMR	BRCA1	splice_acceptor_variant	18	c.5153-1G>A	splice	18%	Likely pathogenic	HRRmt	5	26	1	0
58	AQ2002000FFP	54	I	pMMR								HRRwt	9	17	1	0

59	AQ2002917FFP	52	III	pMMR	BRCA1	frameshift_variant	10	c.981_982del	p.Cys328fs	65.70%	Pathogenic	HRRmt	9	12	0	0
60	AQ2104533FFP	64	IV	pMMR								HRRwt	32	57	1	0
61	AQ2105238FFP	56	II	pMMR								HRRwt	2	8	0	0
